# Analysis of intermunicipal journeys for cardiac surgery in Brazilian Unified Health System (SUS): an approach based on network theory

**DOI:** 10.1186/s12939-023-01857-y

**Published:** 2023-03-16

**Authors:** Ludmilla Monfort Oliveira Sousa, Hernane Borges de Barros Pereira, Edna Maria de Araújo, José Garcia Vivas Miranda

**Affiliations:** 1 Bahia State University, Camaçari, Brazil; 2 SENAI ClMATEC University Center, Salvador, Brazil; 3 State University of Feira de Santana, Feira de Santana, Brazil; 4 Federal University of Bahia, Salvador, Brazil

**Keywords:** Management tool, Intermunicipal network, Network analyses, Cardiovascular surgery, Healthcare services

## Abstract

**Introduction:**

The transformation of data into information is important to support decision making and, thus, to induce improvements in healthcare services. The regionalized organization of healthcare systems is necessary to ensure the integrity of citizen care. From this perspective, the creation of mechanisms to guide and assess the behavior of a healthcare services network becomes necessary. However, these mechanisms must consider the interaction between different municipalities. The objective of this study is to apply network analysis as a supporting tool in the Brazilian Unified Health System (*Sistema Único de Saúde*—SUS) management.

**Methods:**

The stages of the proposed method are described and applied in a real situation, analyzing the intermunicipal interaction network for cardiovascular surgery in the municipality of Vitória da Conquista, Bahia, Brazil, from 2008 to 2020. The metrics analyzed were journeys indices, flow of patients and distance of the journeys, considering the journeys from and to the municipality in focus.

**Result:**

There was an increase of the incoming flow and in-degree indices combined with a decrease in outgoing flow, showing the growing importance of this municipality as a provider of these services.

**Conclusion:**

The method used in the study has potential to be adopted as a management tool to assess the behavior of the interactions network of the selected service, aiding the regionalized organization of the healthcare system.

## Introduction

In Brazil, various databases that can be used by healthcare managers to support decision making are freely and openly available [1]. However, these secondary data sources are underused, and some of the reasons are absence of informational culture and of trained personnel to work with the data [2]. Professionals that work in healthcare management can benefit from the incorporation of information from such databases in managing [2, 3]. On this account, developing mechanisms to facilitate the transformation of data into information and then using them in healthcare monitoring and assessment, for decision making, can potentialize healthcare managers’ actions.

The Brazilian public healthcare system, known as SUS (Sistema Único de Saúde—Unified Health System), needs to ensure integral assistance to citizens, thus, when necessary citizens must be referred to other healthcare units, including to other municipalities. That way, healthcare institutions that comprise SUS’s services network should be decentralized and regionally organized [4, 5] composing a hierarchical network that can articulate from the simplest units to the most complex ones, through a system of reference and contra reference to different degrees of complexity. In Brazil, this healthcare regionalization is done with a division of the states’ territories into Healthcare Regions [6, 7].

For building a regionalized and hierarchical network, the responsibility for managing and SUS financing are shared between the three levels of government, namely: the Union, the states, and the municipalities. However, the SUS has as one of its organizational principles the decentralization of management and public health policies, which, in turn, have the purpose of transferring responsibility and resources to municipalities. In this way, municipal managers are encouraged to be attentive to the needs of the population residing in the municipality in which they operate and to offer the appropriate health services. However, most municipalities are not able to provide all health services, and that is why the regionalization of these services is so important to guarantee the integrality of care for citizens. And to build this regionalized network of health services, there are the commissions of managers between levels of government, which are intergovernmental spaces for planning, negotiating, and implementing public health policies [6, 7].

Network approaches are used in decision-making processes to improve healthcare efficiency. The network analysis was also employed as one of the processes to constructing models to be used in healthcare [8, 9]. Some studies were applied for prediction and diagnosis objectives [10], medical effect prediction [11], improvement of medicine distribution logistics [12], and other reasons.

For this reason, creating mechanisms to assess and guide the intermunicipal networks that provide citizens with access to healthcare services is necessary, considering the interrelation between municipalities, and verifying how a location influences and is influenced by those around it. The Hospital Information System (*Sistema de Informação Hospitalar*—SIH-SUS) enables the verification of the municipality in which SUS patients live and the municipality in which they are hospitalized [13], thus enabling the analysis of the incoming and outgoing flows of patients for hospitalizations. In this way, the municipal manager would be able to carry out the following analyses: where do more patients come from for the municipality under my management? Where do patients residing in the municipality under my management go? Do they make big displacements? Could it be that with this volume and the distance covered, would it not be better to offer certain services in strategically located municipalities? How to regionalize health services to offer them more efficiently and safely to patients? Patients' incoming and outgoing flow is a phenomenon that can be modeled with a network, enabling the analysis of how this network is organized.

In general terms, relationships between entity pairs can be represented through networks. Mathematically, a network is represented by a graph. A graph is composed of a set of vertices (or nodes, or points), and edges or arcs (or links, or lines). If two vertices are linked by a line it means that they are connected because they keep some relation of interest. In building a network it is possible to study its entities from a local, global, topological, temporal and spatial perspective and it is possible to investigate the mechanisms of interaction between the components that form the system [14–19].

Aiming at supporting decision making in healthcare systems, specifically in the assessment of SUS patients intermunicipal flow, the objective of the study reported in this article is to propose a method for the analysis of intermunicipal journeys for hospitalizations in SUS, using network analysis as a management tool for SUS. To describe the application of the method, the networks of cardiovascular surgeries hospitalization, which represent a high complexity healthcare service, were investigated.

## The proposed method

In this article we describe the network analysis method to analyze SUS patients’ intermunicipal journeys for healthcare services. Similar analyses had been conducted in other studies [20, 21], however, these studies focus on analysis at the State level. Moreover, the incoming and outgoing flows of patients have been used to analyze citizens' access to health services and strengthening of regionalization. Initial studies have already demonstrated this type of displacement in general in Brazil [22, 23] and focused on these types of patient flows. More recent research demonstrated the incoming and outgoing flows and added some other network indices to perform this analysis [20, 21, 24, 25].

The difference of this article is the application of the network analysis method at the municipality level, with a detailed description of the method, so that it can be replicated by healthcare managers and other professionals working with healthcare data. After all, the positive impacts observed in State and National data is a result of the organization of the healthcare system in the municipalities. This method can be used for different types of healthcare systems.

**Fig. 1:**
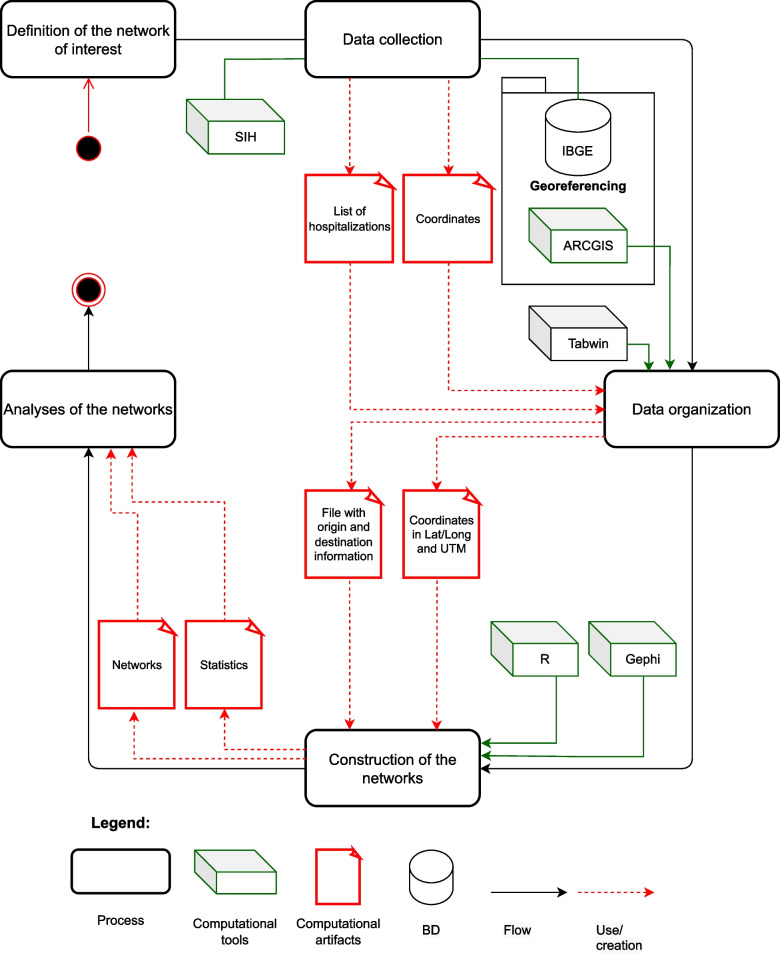
Flowchart describing the proposed method for network analysis

To detail the proposed method, the flowchart (Fig. 1) describes all the steps followed in this study.

### Definition of the network of interest

There are various Healthcare Information System (Sistema de Informação em Saúde—SIS) with many different databases that provide us an infinitive of health data with large potential. This variety of databases, such as the Hospital Information System (*Sistema de Informação Hospitalar*—SIH), Ambulatorial Information System (*Sistema de Informação Ambulatorial*—SIA), Minimum Data Set (*Conjunto Mínimo de Dados*—CMD), among others, can be used in network analyses.

First, it is necessary to define which healthcare service to analyze, because the ideal spatial distribution of the healthcare units in the territory may be different according to the type of service under study. It is also important to know if the information system used has the necessary data to generate a network. The system must have at least the municipality of origin and the destination of SUS patients.

To demonstrate the application of the network theory in the present study, the journeys for intermunicipal cardiac hospitalizations were analyzed, thus representing a high complexity modality of hospital care. Cardiac problems deserve emphasis because of the relevance of cardiovascular disease in the national scenario, considering that it is the main cause of death in Brazil [22–29] and the cause of hospitalizations with the highest expenses.

**Fig. 2:**
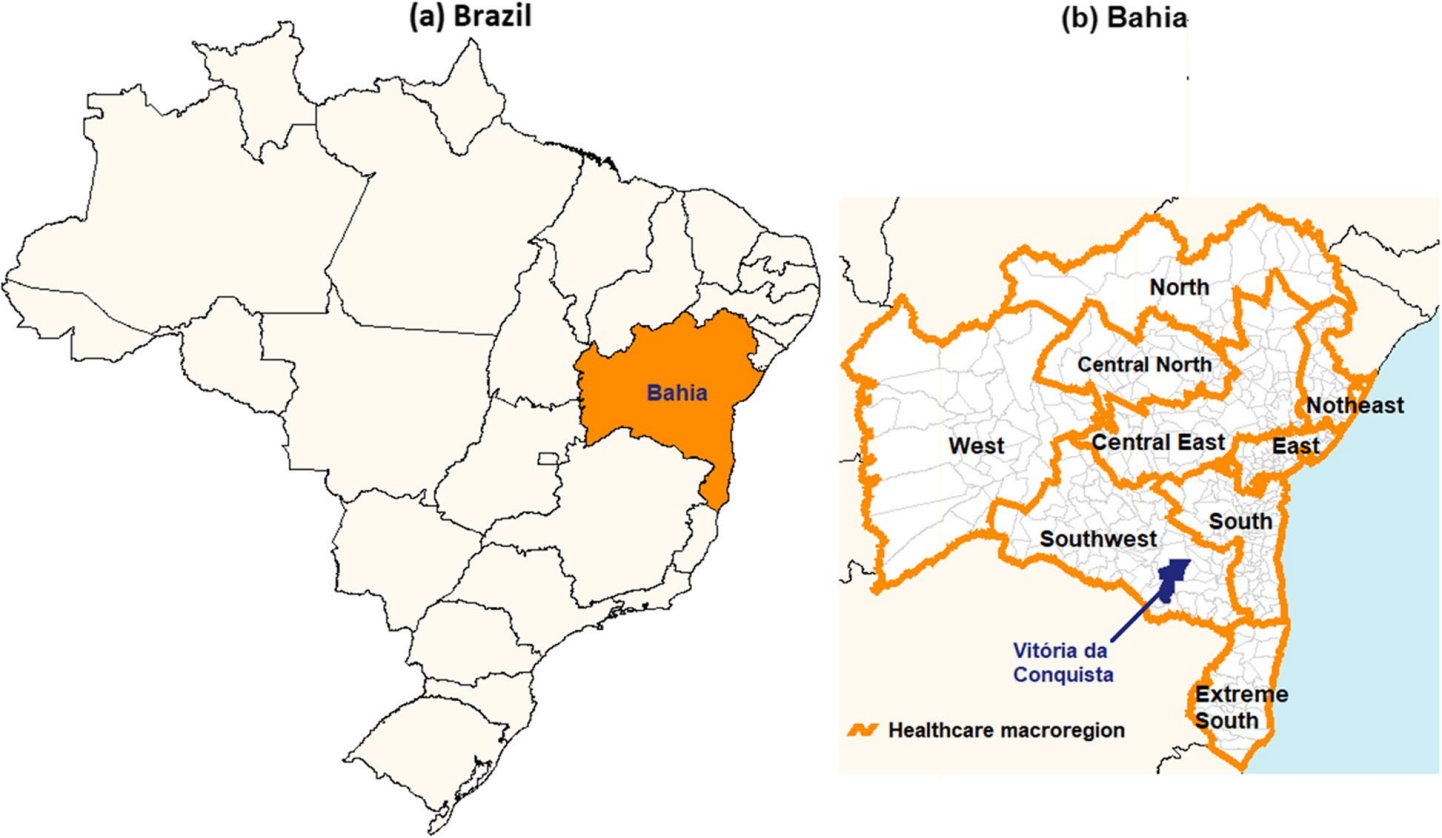
**a** Spatial location of Bahia in Brazil. **b** Spatial location of Vitória da Conquista in Bahia

Only one municipality was selected to facilitate the demonstration of the proposed method. Therefore, the results found in one municipality may be different in other municipalities. Within this context, it is important to analyze the results and contextualize them in the territory where the information was recorded, because what happens in one territory can be different in another territory. To demonstrate the cardiovascular surgery network, the municipality of Vitória da Conquista [26], Bahia, Brazil, was selected. This municipality is located in the Southwest macroregion of the state of Bahia (Fig. 2), being the municipality of reference of the macroregion that showed the highest decrease in the number of cardiovascular surgeries as well as the lowest proportion of municipalities without records about cardiovascular surgery.

### Data collection

To construct the networks, it is necessary to prepare two types of files, one for the arcs, and other for the vertices. The file with the arcs contains the patients' journeys, from the municipality of origin to the destination in which the procedure would be performed. The file with information about the vertices contains the spatial locations of the municipalities in the territory that will be analyzed. The documents and databases are on a server which can be accessed by the URL https://doi.org/10.5281/zenodo.7470351.

#### SIH / List of hospitalizations

To create the file containing the arcs, data from the Hospital Information System (SIH/SUS) were used. The SIH/SUS is the system in which the records of the patients hospitalized in the units that are part of SUS (public or associated units) are processed.^1^ These units send hospitalization information collected through the Hospitalization Authorization Form (*Autorização de Internação Hospitalar*—AIH). After data is sent to Datasus, SUS Department of Informatics, this information becomes part of the national database, for further dissemination [13]. These files about the AIH are generated monthly by Brazilian municipalities. In the present study the files from Bahia (RDBA) from 2008 to 2020 were downloaded. The downloaded files are organized in the format RDBAaamm.dbc, in which “aa” is the year of the file and “mm” is the month. The variables “municipality of origin” and “destination” are some of the information available in the hospitalization list generated by the SIH/SUS, which are necessary to analyze the flow of patients.

#### INDE / Coordinates

Data of the National Infrastructure of Spatial Data (*Infraestrutura Nacional de Dados Espaciais*^2^ - INDE) were used for the creation of the file containing the information about the network vertices. This web page makes geospatial data available through a network of servers connected to the internet. This web page provides files of type “shape” for download of the cities in the 417 municipalities in Bahia. To do so, the option “*Geo serviços*” (Geo services) was selected, then the INDE visualizer (VINDE), then “*Adicionar camadas*” (Add layers) in the option “*Temas*” (Themes). In this stage, two maps were selected: one to get the location of the city of Salvador and the other to get the locations of the cities in Bahia municipalities. The map containing Brazilian capitals (*BCIM Capital—Ponto*) was used to get Salvador’s location and the map of cities (*BCIM Cidade—Ponto*) was used to get the locations of the other cities in Bahia municipalities.

### Data organization

#### List of municipalities of origin and destinations

The intermunicipal journeys by SUS patients were organized as matrices of municipality of origin and destination. This matrix is analogous to the matrix of migration, or cost matrix, used to represent a graph/network. In this matrix, the municipalities in Bahia were organized in a way so that the lines contained the municipalities of residence (municipalities of origin) and the columns contained the municipalities of occurrence of the group of procedures under study. The matrices of origin and destination were constructed with the software Tabwin [30].

To generate the matrix of cardiovascular surgery, SUS codes of procedures listed in the Covenanted and Integrated Plan (*Programação Pactuada e Integrada*—PPI) about cardiovascular surgery were filtered, in the intermunicipal hospital plan of high complexity [31], along with the municipalities of residence and occurrence in Bahia. To visualize the temporal evolution of the municipalities’ intermunicipal networks, a matrix of origin and destination was generated for each year. Subsequently, these data were reorganized so that they could be imported in a network analysis tool. In this study, the tool used was Gephi. Each matrix of origin and destination generated another file, here called list of origin and destination, containing the columns origin, destination and weight. In this newly generated file, the origin column is the municipality of residence, the destiny column is the municipality in which the procedure was conducted, and the weight column represents the number of people that traveled from the municipality of residence to the municipality of the procedure.

#### Coordinates in Lat/Long and UTM

The software ArcGis was used to reorganize the data for the construction of the file with the vertices of the network. The file with the vertices was generated to contain two different formats of coordinates: (1) latitude and longitude that must be in decimal degrees (lat/long), and (2) coordinates in UTM (Universal Transverse Mercator). To do the spatialization of the municipalities’ cities in Gephi, the coordinates used were in lat/long, and to calculate the mean length of the arcs it is important that the coordinates used are in UTM.

### Construction of the networks

With the data organized, two types of report were generated with the software Gephi and R.

With Gephi, the spatial distributions of the networks were generated and the network indices were calculated, which in this study were called migration indices, as they are linked to people's journeys. The two migration indices generated were in-degree and out-degree. With R, four statistical indices were generated, namely: incoming flow, outgoing flow, mean length of the incoming arcs and mean length of the outgoing arcs. Based on the indices generated from the networks’ spatial distributions, it is possible to characterize the hospitalization network of a municipality and to know how many people and from how many different municipalities people are looking for healthcare services in the municipality under analysis. On the other hand, it is also possible to verify how many people are traveling out of the municipality, the municipality from which they are traveling and the number of different municipalities to which they are traveling for procedures.

### Analyses of the networks

The intermunicipal journeys organized as networks can be considered as a directed graph. A graph is a structure $G=(V,A)$ in which $V$ is the set of vertices or nodes and $A$ is the set of arcs of the network, considering that the networks in this study are directed [14].

The indices used in the analyses of the networks in this study were: in-degree, out-degree, incoming flow, outgoing flow, mean length of incoming arcs and mean length of outgoing arcs [20, 21].

In the present study, the in-degree index is the number of arcs coming into a vertex (${k}_{I}$). In this study, the interpretation of this index is similar to the immigration of patients to a given municipality. The municipality in which the procedure is conducted is the reference, thus this index quantifies the number of different municipalities from which people traveled in search for a procedure in the municipality under analysis. The out-degree is the number or arcs going outwards of a vertex (${k}_{O}$), in this study its interpretation is similar to the emigration of patients, representing the municipality of residence and thus quantifies the number of different municipalities to which people travel in search of the procedure. The incoming flow is the weight of the incoming arcs of a vertex (${F}_{I}$). The interpretation of this index in this study is the number of people that arrive at a municipality for the procedure in question. The outgoing flow is the weight of the outgoing arcs (${F}_{O}$), and in this study it measures the number of people that traveled out of their municipality of residence for a procedure in another municipality.

The mean length of the outgoing arcs, initially presented by Sousa et al. (2017) [20] and used in Sousa et al. (2020) [21] is represented by Eq. 1:

1 $$\overline D=\frac1{\sum_{a=1}^{k_0}F_a}\sum_{a=1}^{k_0}D_aF_a$$

in which ${k}_{O}$ is the out-degree, ${D}_{a}$ is the distance of the journey, in kilometers, from the municipality of residence to the destination municipality, and ${F}_{a}$ is the number of people that traveled.

The equation above represents the weighted average of the distances between the municipalities in which outgoing flow of patients were observed [20]. It is important to emphasize that this index considers the straight-line distance, in other words, the shortest distance between two municipalities, with the possibility of longer real distances depending on the road layout and the means of transportation used. Notwithstanding, considering the processes of analysis and decision making, this index turned out to be appropriate to measure the distances that citizens are traveling to have access to the healthcare service in question.

Finally, the mean length of the incoming arcs, whose value represents the weighted average of the distances between the municipalities in which incoming flow of people for the procedure under analysis was observed, is similar to Eq. 1, replacing the out-degree (${k}_{O}$) with the in-degree (${k}_{I}$).

## Results

**Fig. 3:**
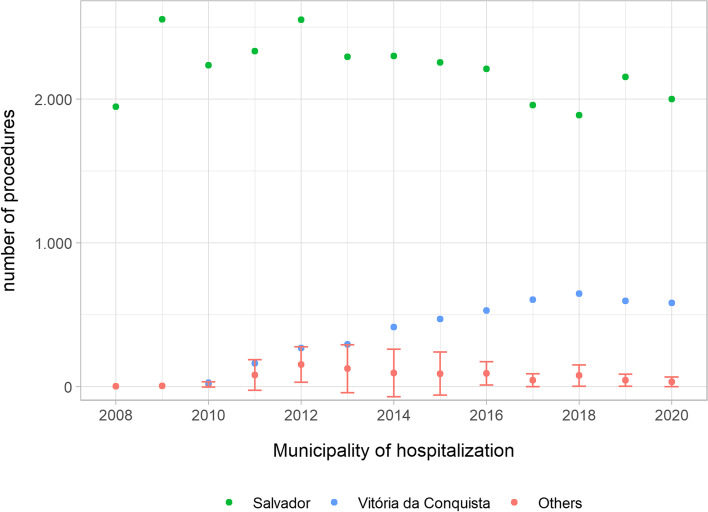
Number of hospitalizations for cardiovascular surgery in Bahia, by service provider municipality, 2008 to 2020. Data Source: SUS Hospital Information System (*Sistema de Informações Hospitalares do SUS*—SIH/SUS)

Applying the proposed method of network analysis to Vitória da Conquista showed that this municipality started conducting cardiac surgery procedures in 2010 with a constant increase in the number of such procedures each year (Fig. 3). From 2015, it became the municipality with the second highest number of this type of surgery in the state of Bahia, only behind Salvador.

**Fig. 4:**
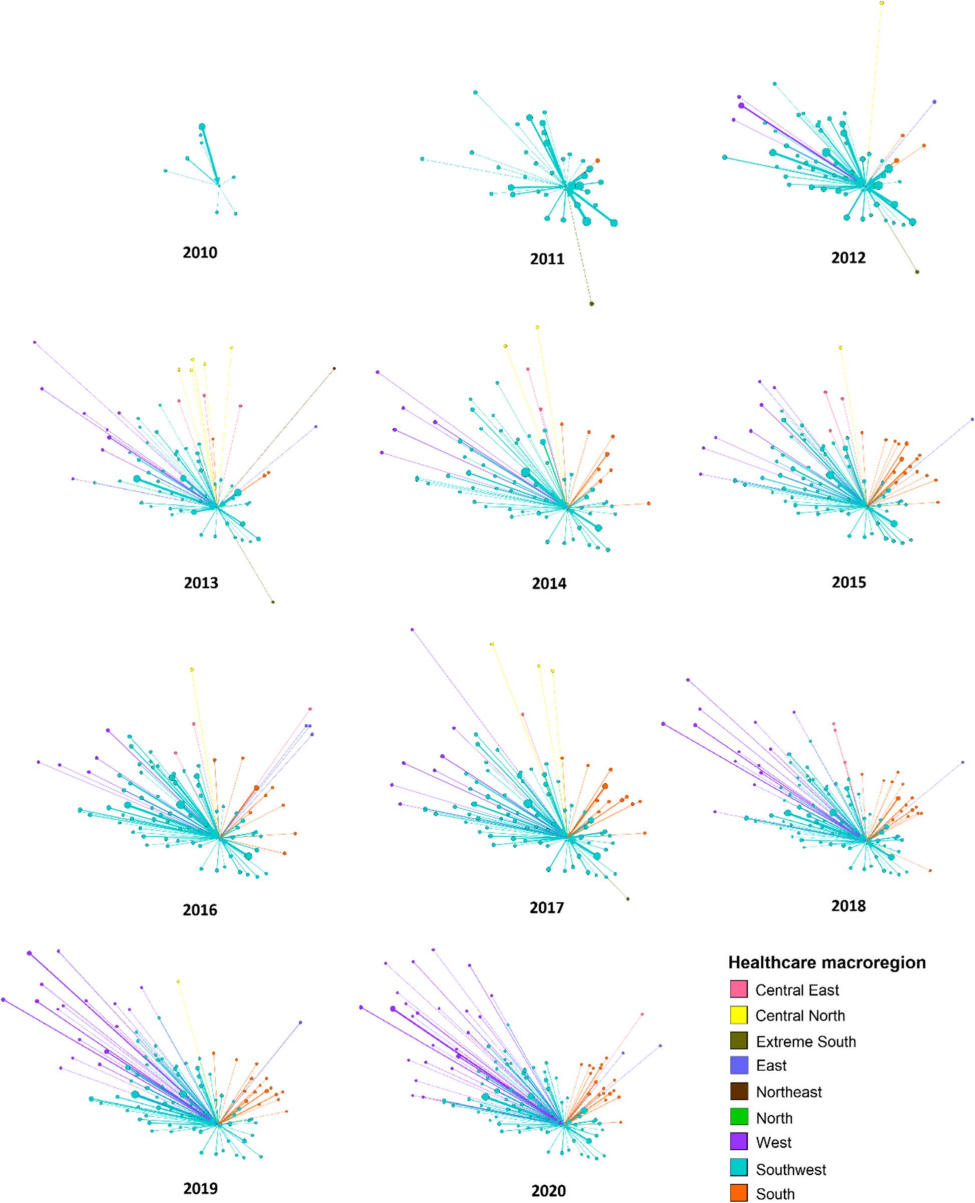
Evolution of Vitória da Conquista intermunicipal network of cardiovascular surgery, vertices geographically localized. Bahia, 2010 to 2020

Figure 4 shows the evolution of the network of intermunicipal hospitalization for cardiovascular surgery in the municipality of Vitória da Conquista. In 2010, there were records of hospitalizations only in municipalities of the Southwest macroregion, and from 2014 hospitalizations of patients from 5 different macroregions were registered.

The indices shown in Table 1 enables the analysis of the evolution of the intermunicipal network of cardiovascular surgery. From the in-degree, it is possible to observe that in 2010 Vitória da Conquista had records of patients coming from 8 different municipalities, and in 2020 this number increased to 115. From these 115 municipalities, 71 (61,7%) were from the same Southwest macroregion, 23 (20%) from the West macroregion and 18 (15,7%) from the South macroregion. In relation to the out-degree, however, in 2012 and 2014 there were records of cardiovascular surgery in Vitória da Conquista citizens in two different municipalities, namely Salvador and Itabuna, and in the other years only in Salvador.

**Table 1:** Indices of the Vitória da Conquista intermunicipal network of cardiovascular surgery. Bahia, 2008 to 2020

Indices	Network indices		Statistical indices			
	**In-degree**^**a**^	**Out-degree**^**b**^	**Incoming Flow**^**c**^	**Outgoing Flow**^**d**^	**Mean length of incoming arcs (km)**^**e**^	**Mean length of outgoing arcs (km)**^**f**^
**2008**	0	1	0	35	NA	326.52
**2009**	0	1	0	43	NA	326.52
**2010**	8	1	13	25	131.57	326.52
**2011**	35	1	66	5	103.33	326.52
**2012**	59	2	162	6	127.25	274.36
**2013**	71	1	183	1	167.57	326.52
**2014**	75	2	257	3	146.33	274.36
**2015**	90	1	307	4	156.54	326.52
**2016**	89	1	362	5	159.52	326.52
**2017**	93	1	398	6	147.80	326.52
**2018**	103	1	467	2	169.32	326.52
**2019**	104	1	445	10	200.45	326.52
**2020**	115	1	456	4	194.98	326.52

*NA* [not applicable]

^a^In-degree: number of different municipalities from which patients traveled for the procedure

^b^Out-degree: number of different municipalities to which patients traveled for the procedure

^c^Incoming Flow: number of patients that arrived at the municipality for the procedure

^d^Outgoing Flow: number of residents that traveled to other municipalities for the procedure

^e^Mean length of incoming arcs (km): mean distance traveled by the patient to arrive at the municipality in which the procedure is conducted

^f^Mean length of outgoing arcs (km): mean distance traveled by the patient to go to the municipality in which the procedure is conducted

The incoming flow measures the number of patients from other municipalities that went to Vitória da Conquista for cardiovascular surgery. Table 1 shows evidence that this number continually increases, since it began in 2010, with 13 hospitalization records, and in 2020 with 456 hospitalization records of patients from other municipalities. The analysis of the outgoing flow enables the observation of a decrease in the number of patients that traveled out of Vitória da Conquista for such procedures in other municipalities from 2010. In 2008 there were 35 hospitalization records of Vitória da Conquista residents in other municipalities, but in 2020 this number decreased to 4 patients.

The mean length of the incoming arcs enables the estimate of the straight-line distance of the journey by SUS patients to the municipality in which the procedure was conducted. The analysis of this index in Table 1 shows that it has been continually increasing. In 2010, this mean distance was about 131 km, but in 2019 the longest distance in the historic series was about 200 km. Regarding the mean length of the outgoing arcs, the distance was about 327 km, but in the years 2012 and 2014 there was a reduction to approximately 275 km.

## Discussion

In this study, Vitória da Conquista was considered increasingly important for the access to cardiovascular surgery in the state of Bahia. The crescent number of hospitalizations for cardiac surgery along the historic series combined with the increase in this type of hospitalization of Vitória da Conquista residents and with the decrease in the outgoing flow suggests that Vitória da Conquista residents do not need to travel to other municipalities to access this healthcare service. Vitória da Conquista is the third largest municipality in Bahia [32] and has the highest gross domestic product (GDP) and the highest increase in this index in the Southwest macroregion [33]. This result is in line with the study about geographical and social inequalities in the access of healthcare services in Brazil [34], which shows evidence that the citizen’s location of residence influences the access to healthcare services, and that this access improves according to the socioeconomic development of the region.

The incoming arcs, or mean distance of the journey to arrive at the municipality in which the procedure is conducted, increased continuously during the historic series. Since there was an increase in the in-degree, the increase in the mean length of the arcs can be a consequence of the increase in hospitalizations of people from other municipalities, especially municipalities from distant macroregions. In 2020, the mean length of the incoming arcs was approximately 195 km. A study that mapped the hospital care network in Brazil [35] observed that only 3% of the patients that were hospitalized for cardiac surgery were residents in municipalities more than 60 km apart from the municipality in which the procedure was conducted, and about 40% of Brazilian population live in those municipalities that are more than 60 km apart from the healthcare service. The regionalization of healthcare services is important to ensure the equity in the access and use of healthcare services, however, the studies suggest that regionalization is still a challenge to overcome. Since distance can be a factor that interferes in the access and use of healthcare service, the definition of the arc length can be an important index in planning the municipalities to which patients traveled in search of the procedure, which serve as municipalities of reference.

In two years, 2012 and 2014, there was a decrease in the mean length of the outgoing arcs, or mean distance of the journey to arrive at the municipality in which the procedure is conducted. This variation occurred according to the increase in the out-degree. In these two years there were records of cardiovascular surgery in Salvador and Itabuna. Since Itabuna is closer from Vitória da Conquista in comparison with Salvador, the mean length of the outgoing arcs decreased reflecting this smaller distance.

Looking at the sharp increase in cardiovascular surgeries in Vitória da Conquista in the period under analysis is important, especially involving patients from municipalities in different macroregions. Because of the Covenanted and Integrated Plan (*Programação Pactuada e Integrada*—PPI) [31], every municipality has defined which other municipality is a reference in conducting a given healthcare service. The data from this method of analysis can support the development of PPI, supporting in the definition of the municipalities of reference in conducting cardiac surgery services in the state of Bahia and, particularly, in Vitória da Conquista.

Despite hospitalizations for cardiovascular surgery being selected in this study, the proposed method can be used to analyze any other type of hospitalization. However, it is relevant not to combine different types of hospitalizations in a single network, since each type of hospitalization can inform different layouts of regionalization.

To facilitate the application of the proposed method, only the hospitalizations in Bahia were considered. However, this method can be used to verify hospitalizations between municipalities in different states.

Secondary data from healthcare information databases available online were used to demonstrate the application of the method. Because of the great volume of data, the availability of information and the easy access to databases about hospitalizations, the use of such information increased in Collective Health [36]. Thus, the use of secondary databases is important in assessment studies, because of their low cost, the wide availability of data, the speed of data collection and results, and for their possible impact in healthcare services [37].

Considering that secondary databases from the Healthcare Information System were used, it was and it is important to verify the reliability of the data used in the system, to make a robust and adequate healthcare planning. From this perspective, the field that records the data “conducted procedure” (“*procedimento realizado*”) has been considered satisfactorily reliable by some authors [38–40]. On the other hand, regarding the field “municipality of residence” (“*município de residência*”), a study that analyzed its quality for woman hospitalized because of breast or cervical cancer in Rio de Janeiro [41] found an accuracy of 82.9% when compared to records from the Information System about Mortality (*Sistema de Informação sobre Mortalidade*—SIM) and SIH/SUS, suggesting high reliability; however, this reliability decreased when a woman lived outside the municipality in which hospitalization was required. Since the proposed method considered only the patients whose municipality of residence was different from the municipality in which they were hospitalized, it is important to consider some limitations, such as the omission of the true municipality of residence. Even with this limitation, the studies conducted in Rio de Janeiro showed enough quality in the field “municipality of residence” (“*município de residência*”) to support the decision-making process in healthcare management [41].

There are various challenges in the construction of a system that must be unified and in compliance with a regionalized network of services [4, 5, 42]. Therefore, the creation of permanent mechanisms of network assessment that can support the construction of regional planning becomes indispensable. Network analysis, as presented in this article, can reveal general aspects of the phenomenon under assessment, and the combination with a qualitative approach is advised, because they are complimentary, enabling the assessment of different peculiarities of the same phenomenon [43].

Since the cutoff of this paper was for only one municipality, the network approach loses its strength. In this case, the same conclusions could have been obtained using a statistical based on the flow databases analysis of the municipalities together with their geolocation. However, the network approach, in addition to simplifying the algorithms for calculating the indices, allowed us to use a schematic view of the temporal evolution of the demand coming from other regions of the state. Future work could extend the proposed approach here to evaluate the topological properties of the network formed by sets of municipalities, characterizing the relationship between the demands of the regions and their temporal evolution.

## Conclusions

The proposed method for monitoring and assessment of the intermunicipal hospitalization network can be used as a management tool to analyze the behavior of this and other networks according to the selected services of interest. Assessment methods are important in the management process, because they provide elements that assist decision making.

The practical application of the proposed method enabled the verification of the number of patients that traveled from their municipality of residence to the municipality in which the service was conducted, as well as the number of different municipalities for which those citizens traveled, and the traveled distance to arrive at the municipality with the objective of having access to healthcare assistance. In this way, from another perspective, it is possible to verify the number of people arriving at a municipality that provides the healthcare service, their municipality of residence, and the distance they travel to arrive at the destination.

The results show evidence of the possible wide use of this methodology to the assessment of the hospitalization network systems. Therefore, the proposed method can assist the planning of an intermunicipal hospitalization network, as well as support the decisions about the best way of regionalizing healthcare services.

The results also suggest that to improve access to this essential medical service in a particular location, regionalization techniques need to be reviewed. To lower the death rates from cardiac surgery, it is essential to guarantee access to high-quality care. Regionalization of care can aid in the concentration of resources and knowledge in specialized health centers, but it must be properly planned to guarantee that all cardiovascular patients, regardless of their location, have equitable access to care. Cardiovascular patients who lack proper access to care may experience unfavorable results, hence it is crucial to address this problem through efficient regionalization policies and initiatives.

## Data Availability

The first datasets generated and/or analyzed during the current study are available in the Datasus repository, https://datasus.saude.gov.br/transferencia-de-arquivos/. The second datasets are available in the INDE repository, http://www.inde.gov.br/. The documents and databases used in this research are on a server which can be accessed by the URL https://doi.org/10.5281/zenodo.7470351.

## Footnotes

1 Data available at: https://datasus.saude.gov.br/transferencia-de-arquivos/#, last access on 12/17/2022 at 5:50 pm.

2 Data available at: https://visualizador.inde.gov.br/, last access on 12/17/2022 at 6:00 pm.
